# *In vivo* characterization of connective tissue remodeling using infrared photoacoustic spectra

**DOI:** 10.1117/1.JBO.23.12.121621

**Published:** 2018-12-05

**Authors:** Yuan Qu, Peng Hu, Junhui Shi, Konstantin Maslov, Peinan Zhao, Chiye Li, Jun Ma, Alejandro Garcia-Uribe, Karen Meyers, Emily Diveley, Stephanie Pizzella, Lisa Muench, Nina Punyamurthy, Naomi Goldstein, Oji Onwumere, Mariana Alisio, Kaytelyn Meyenburg, Jennifer Maynard, Kristi Helm, Emma Altieri, Janessia Slaughter, Sabrina Barber, Tracy Burger, Christine Kramer, Jessica Chubiz, Monica Anderson, Ronald McCarthy, Sarah K. England, George A. Macones, Molly J. Stout, Methodius Tuuli, Lihong V. Wang

**Affiliations:** a Washington University in St. Louis, March of Dimes Prematurity Research Center, Department of Obstetrics and Gynecology, St. Louis, Missouri, United States; b Washington University in St. Louis, Department of Biomedical Engineering, St. Louis, Missouri, United States; c California Institute of Technology, Caltech Optical Imaging Laboratory, Andrew and Peggy Cherng Department of Medical Engineering and Department of Electrical Engineering, Pasadena, California, United States

**Keywords:** photoacoustic endoscopy, spectroscopy, cervical examination, tissue hydration

## Abstract

Premature cervical remodeling is a critical precursor of spontaneous preterm birth, and the remodeling process is characterized by an increase in tissue hydration. Nevertheless, current clinical measurements of cervical remodeling are subjective and detect only late events, such as cervical effacement and dilation. Here, we present a photoacoustic endoscope that can quantify tissue hydration by measuring near-infrared cervical spectra. We quantify the water contents of tissue-mimicking hydrogel phantoms as an analog of cervical connective tissue. Applying this method to pregnant women *in vivo*, we observed an increase in the water content of the cervix throughout pregnancy. The application of this technique in maternal healthcare may advance our understanding of cervical remodeling and provide a sensitive method for predicting preterm birth.

## 1 Introduction

The cervix is a remarkable structure with diametrically opposite functions: it maintains pregnancy by remaining closed and then, in a process called remodeling, softens and dilates to allow delivery of the fetus in labor.^1^ Premature cervical remodeling is a critical indicator of impending spontaneous preterm birth. Preterm birth can occur with a remodeled cervix even in the absence of uterine contractions, but uterine contractions do not lead to delivery if the cervix is firm.^2^^–^^4^ Nevertheless, current clinical measurements of cervical remodeling are largely obtained by digital examinations, which are subjective and detect only late events, such as cervical effacement and dilation.

The cervix remodels progressively via incompletely understood mechanisms, such as degradation of extracellular matrix proteins and inflammation.^5^^,^^6^ These physiological changes are associated with increased tissue hydration.^7^^,^^8^ Therefore, a method that can accurately measure cervical hydration during pregnancy has the potential to facilitate our understanding of cervical remodeling and permit more accurate prediction of preterm birth.

Near-infrared spectroscopy is routinely used in industrial applications to quantify the water content in various products, because this method is nondestructive and does not require sample preparation.^9^^,^^10^ As an embodiment of near-infrared spectroscopy, spectroscopic photoacoustic tomography has been demonstrated in the quantification of various biochemical constituents.^11^^–^^14^ However, the previous applications used tabletop systems, which precluded *in vivo* use in the gastrointestinal tract and urogenital tract. Photoacoustic endoscopy (PAE) incorporates an acoustic detector, optical components, and electronic components in a millimeter-diameter-scale probe to image tissue that is inaccessible by tabletop systems.^15^^–^^20^

For the quantification of the water content of the cervix in a pregnant woman, the combination of PAE and near-infrared spectroscopy provides an optimal solution. However, the task is nontrivial, because PAE needs an acoustic coupling medium, which generally contains water as well. The photoacoustic signals emitted by the acoustic coupling medium are not easily separable from the signals emitted by the tissue in the near-infrared wavelength range. This challenge so far has precluded the use of near-infrared spectroscopic PAE for the quantification of water content.

Here, we present a near-infrared spectroscopic PAE system that transmits acoustic waves from the tissue to the acoustic detector through an N-BK7 pentaprism. We analyze the measured photoacoustic near-infrared (PANIR) spectra by linear regression. We demonstrate that this method successfully quantifies the water contents of tissue-mimicking phantoms made of gelatin hydrogel. Applying this method to the cervices of pregnant women, we observe their physiological water contents and a progressive increase throughout gestation.

## 2 Methods

### 2.1 System Setup

**Fig. 1:**
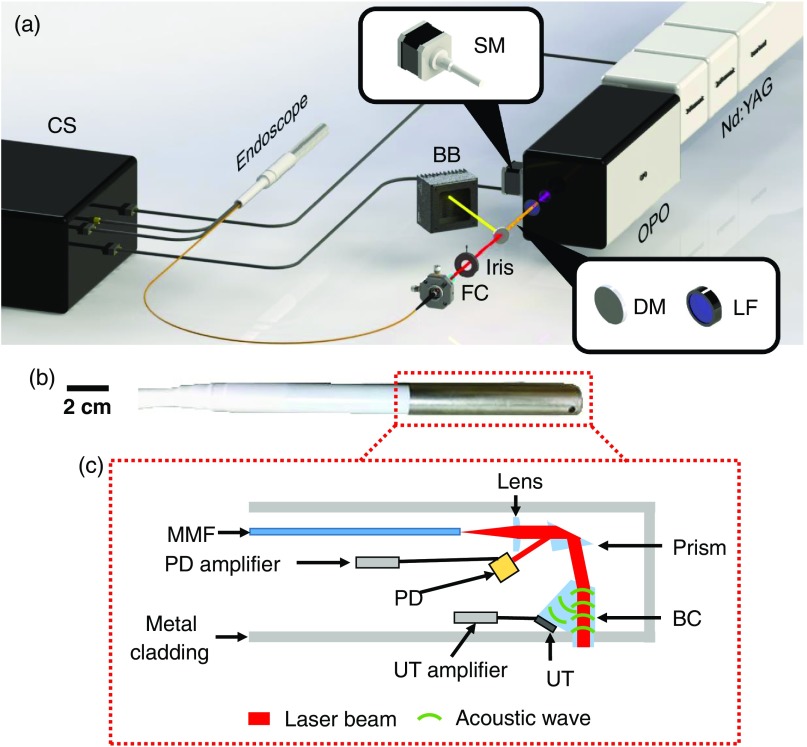
PANIR system. (a) Setup of the PANIR system. BB, beam block; CS, control system; DM, dichroic mirror; FC, fiber coupler; LF, longpass filter; Nd:YAG, Nd:YAG laser with a frequency tripling module; OPO, optical parametric oscillator; SM, stepper motor. (b) Photograph of a PANIR probe. (c) Schematic of the components in the probe. BC, beam combiner; MMF, multimode fiber; PD, calibrated photodiode; UT, ultrasonic transducer.

We developed the PANIR system shown in Fig. 1(a). The system is controlled by a custom-designed program written in LabVIEW (National Instruments). A frequency-tripled Nd:YAG laser (Quantel, Q-smart 450), operating at 355-nm wavelength with a 20-Hz pulse repetition rate, pumps an optical parametric oscillator (GWU-Lasertechnik, basiScan). A stepper motor moves the optical parametric oscillator so that the idler light can be scanned from 1000 to 2000 nm. After passing through the oscillator, the remaining energy of the pump light is absorbed by a longpass filter. The idler light is selected by a dichroic mirror and then coupled into a multimode fiber, which guides the light to the PANIR probe [Fig. 1(b)]. An iris between the dichroic mirror and the fiber coupler controls the delivered optical energy, keeping the optical fluence (mJ/cm^2^) on the tissue surface below the American National Standards Institute safe exposure limit.^21^

The internal structure of the PANIR probe, which is 30 cm in the length and 2 cm in the diameter, is shown in Fig. 1(c). The idler light from the multimode fiber is projected onto the tissue surface by a plano-convex lens and a prism and is absorbed by the tissue below the optical-acoustic beam combiner—a custom-designed pentaprism. The backward photoacoustic wave propagates through the beam combiner toward an ultrasonic transducer (2.25-MHz central frequency). The configuration of all these optical and acoustic elements reduces the amount of light absorbed by the ultrasonic transducer to a negligible level. While the idler light is sweeping over the entire spectral range, the detected photoacoustic signal is always overwhelmed by noise when only air is underneath the beam combiner. The InGaAs photodiode (FD10D, Thorlabs) in the probe continually measures the energy of the idler light to correct for its energy fluctuations in subsequent data processing. Furthermore, every day, we calibrate the PANIR system with graphite to correct for instrument drift.

### 2.2 Human Studies

Participants were recruited from the patient population attending the Obstetrics and Gynecology Clinic and the Women’s Health Center in the Barnes-Jewish Hospital Center for Outpatient Health. Eligibility requirements included an age of 18 or older, the capability of informed consent, and a gestational age of <16  weeks. Exclusions included potential participants who were non-English speaking, unwilling to participate, carrying a twin pregnancy, or showing evidence of major fetal anomalies.

Prior to measuring the cervix, the operator placed a speculum in the vagina, exposing the cervix for PANIR measurements. The PANIR spectrum has a spectral resolution of 5 nm and a scan of one spectrum takes 10 s. All experimental procedures were carried out in accordance with the protocols approved by the Institutional Review Board of Washington University in St. Louis. All participants signed informed consents before inclusion in the study.

## 3 Results

### 3.1 Phantom Experiments

**Fig. 2:**
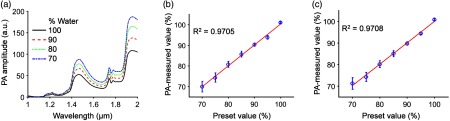
PANIR spectra quantify the hydration of hydrogel. (a) PANIR spectra measured from hydrogel phantoms made of water and gelatin with different fractions. (b) and (c) Water contents measured at (b) 1460 nm and (c) 1940 nm versus the preset values.

We first quantified the water content in phantoms made of hydrogel because of its similarity to connective tissues.^22^^–^^24^ In phantom preparation, a beaker filled with a mixture of gelatin and distilled water was placed on a hot plate and heated to 90°C. A stir bar stirred the mixture at a constant speed. After the gelatin powder was completely dissolved in the mixture, we let the mixture solidify in a Petri dish at room temperature (20°C). When we measured the PANIR spectrum of the phantom, it was kept at 37.5°C to mimic the temperature of the human cervix, and its weight was measured every hour to track the change of water content due to evaporation. Figure 2(a) shows typical phantoms’ PANIR spectra, which move upward as the water content decreases.

To quantify the water content, we fitted a single-wavelength linear regression model based on the empirical calibration because the intercorrelation effect of multiwavelength models led to strong instability.^9^ To minimize the correlation between our measurements and any variation in the environment, we collected PANIR spectra by random sample selection for both the calibration set and the validation set. The calibration set and the validation set each included 350 PANIR spectra, measured from phantoms whose compositions covered the entire range of water contents in soft human tissues (70% to 100%).^25^ We tested our method at two wavelengths, 1460 nm, corresponding to the first overtone of O─H stretching, and 1940 nm, corresponding to the second overtone of O─H bending.^26^ Figures 2(b) and 2(c) show the results and confirm that the measurements of water content agree with the preset values. As the water content in the hydrogel decreased, the standard deviation of our measurements increased, because the gel network became more heterogeneous,^27^ causing the local water content to fluctuate. At either wavelength, the model provided high and similar prediction accuracies.

### 3.2 Human Studies

**Fig. 3:**
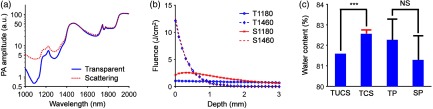
Effects of scattering simulated by the Monte Carlo method. (a) Effect of scattering on the spectrum. We measured the PANIR spectrum of distilled water (blue) and calculated its spectrum distorted by scattering (red), comparable with the degree found in human skin. (b) Effect of scattering on the distributions of fluence in a medium. The absorption at 1460 nm is so strong that the scattering leads to only a small perturbation of the distribution of fluence. S1180, simulated at 1180 nm in the scattering medium; S1460, simulated at 1460 nm in the scattering medium; T1180, simulated at 1180 nm in the transparent medium; T1460, simulated at 1460 nm in the transparent medium. For illustrative purposes, T1460 and S1460 are divided by a factor of two. (c) Quantified water contents for the human tissue and the hydrogel phantoms. The transparent model underestimates the water content of the scattering medium by ∼1%. The red error bar and the black error bar, respectively, show the standard deviations contributed by the cross-sectional change of scattering among the tissue samples (n=16)^30^ and by the heterogeneity of the hydrogel phantoms (n=10). SP, scattering phantom; TCS, tissue corrected for scattering; TP, transparent phantom; TUCS, tissue uncorrected for scattering. ***, P<0.001. NS, nonsignificant.

For human cervical tissue, we must consider the effect of scattering, which distorts the PANIR spectrum.^28^ To understand this influence, we compared our measurements with the results from a Monte Carlo simulation^29^ that used the optical properties of human tissue.^30^ Figure 3(a) shows the distortion of the water spectrum by scattering, comparable with the degree found in human skin. In the wavelength range of 1000 to 1300 nm, where the absorption coefficient ($\mu_{a}\leq 1\;{\mathrm{cm}}^{-1}$) was smaller than the reduced scattering coefficient ($\mu_{s}^{\prime }\approx 12\;{\mathrm{cm}}^{-1}$), the PANIR spectrum was raised [Fig. 3(a)] because more photons were absorbed by water than transmitted [Fig. 3(b)]. However, in the neighborhood of 1460 nm, the absorption coefficient ($\mu_{a}\approx 28\;{\mathrm{cm}}^{-1}$) was so large that the scattering ($\mu_{s}^{\prime }\approx 11\;{\mathrm{cm}}^{-1}$) caused only a small perturbation in the distribution of fluence [Fig. 3(b)]. As a result, the scattering has little influence on the amplitudes in this neighborhood [Fig. 3(a)]. Without correcting the PANIR spectrum for scattering, the water content of a scattering medium would be underestimated, but only by ∼1% [Fig. 3(c)]. This underestimation can be neglected as long as the typical change of water content in a physiological process is much >1%. In addition, the standard deviation of water contents caused by the cross-sectional change of scattering among the tissue samples was about one order smaller than the underestimation.

Furthermore, we compared the simulation to a phantom experiment in which we made one transparent phantom (hydrogel with 18% gelatin) and one scattering phantom (hydrogel with 1% Intralipid and 17% gelatin). The linear regression model underestimated the water content by ∼1% in the scattering phantom, where the reduced scattering coefficient approximated the values used in our simulation. The underestimations in the phantom experiment and in the simulation were consistent. Meanwhile, the heterogenous gel network resulted in a larger standard deviation of water contents in the measurement, in comparison with the cross-sectional change of scattering in the simulation. These results suggest that scattering will have a minor effect, and the heterogeneity of human tissue will dominate the variation of measured water contents in application.

**Fig. 4:**
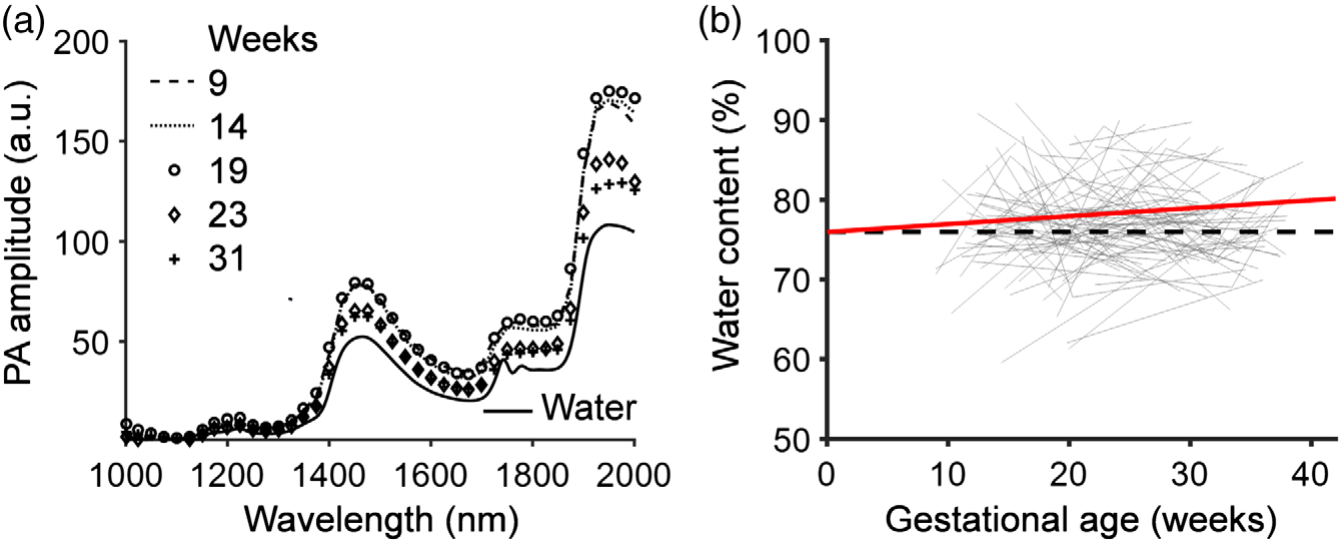
PANIR spectra quantify cervical remodeling. (a) PANIR spectra measured from the cervix of a pregnant woman at five gestational time points. (b) Longitudinal changes of water contents in the cervices of pregnant women (n=205), shown as gray lines. The red solid line indicates the fit at the unit level of the generalized linear model to the measured data. The black dashed line represents the level of intercept.

We validated this method in serial and cross-sectional human studies (Fig. 4), based on the assumption that the hydrogel and the cervical connective tissue were so similar that the regression model derived from one could be applied to the other.^22^^–^^24^ Figure 4(a) shows the PANIR spectra of a pregnant woman at five gestational time points. The PANIR spectra of the cervix showed little change before 20 weeks’ gestation and then dropped to a lower level at the end of the second trimester. We found that the water content increased overall with advancing gestational age [Fig. 4(b)]. The trajectories of water contents, however, were not the same for all patients. We also noticed that the distribution of water contents calculated from the regression model in our study was consistent with the biochemical study.^31^ Furthermore, we carried out a generalized linear model analysis^32^ to assess the linear association between gestational ages and water contents [Fig. 4(b)]. The data for each patient were grouped and modeled as the random component. The gestational age was the irregularly spaced time variable. The slopes calculated in the analysis (Table 1) indicated that the water content had a significant linear effect with respect to gestational age.

**Table 1:** Results of the generalized linear model analysis.

	Value	Standard deviation	Degree of freedom	t-value	p-value
Intercept	76.0 (%)	1.1 (%)	204	67.3	0
Slope	0.1 (%/week)	0.1 (%/week)	147	2.2	0.03

## 4 Conclusions

We have developed *in vivo* PANIR endoscopy that measures the cervical PANIR spectra of pregnant women. With this new technique, we observed serial and cross-sectional changes in PANIR spectra and cervical hydration in pregnancy. Moreover, the measured cervical hydration was consistent with empirically measured values.^31^ Measurement of PANIR spectra and the cervical hydration levels by our system introduces new possibilities for studying preterm birth. They have the potential to explain how environmental or patient-specific factors increase the risk of preterm birth.^33^^–^^36^

Further research and development of our technology could include direct analysis of PANIR spectra using comprehensive machine learning models, which might reveal other phenomena latent in the spectra beyond human perception.^37^^,^^38^ In addition, the reconstructed PANIR spectrum was a mean spectrum from the area under the beam combiner because the photoacoustic signal was detected by a single-element transducer. As a result, the current lateral spatial resolution is ∼2.5  mm. Employing a transducer array and photoacoustic computed tomography^39^^–^^42^ may enable mapping the PANIR spectrum over the same area with a 100-μm spatial resolution. Other optical methods for quantifying cervical remodeling in pregnant women are being developed.^43^^–^^47^ Comparing all optical methods in a large-scale preclinical study would advance our understanding of cervical remodeling from multiple aspects and maximize the prediction accuracy of premature cervical remodeling and preterm birth.
